# From the Global North to the Global South: preparing students for away rotations

**DOI:** 10.1186/s12909-023-04085-8

**Published:** 2023-02-09

**Authors:** Riccardo Serraino, Darius Owachi, Susan Nassaka Byekwaso, Catherine Misango Namara, Kennedy Naigambi, Francesco Castelli, Carlo Torti

**Affiliations:** 1 “Magna Graecia” University of Catanzaro, Catanzaro, Italy; 2 College of Health Sciences, Makerere University of Kampala, Kampala, Uganda; 3 UNESCO Chair “Training and Empowering Human Resources for Health Development in Resource-Limited Countries”, University of Brescia, 25123 Brescia, Italy

**Keywords:** International medicine, Medical education, Global health, Global world, Training

## Abstract

Makerere University College of Health Sciences, Kampala, Uganda, has established partnerships with several other institutions worldwide, including the University of Brescia and “*Magna Græcia*” University, which have agreed to collaborate for the primary purpose of student exchange. Our aim is to comment on students’ preparation for away rotations based on the authors’ own experiences and opinions alongside a review of selected papers on the preparation of students for global health and ethical collaboration. Medical electives represent a unique opportunity for all medical students, not merely for those who will work in resource-limited settings due to increasing globalization. The emergence of ethical international collaborations is of paramount importance to stimulate these projects and ensure that they are implemented safely and with adequate preparation even and especially during the COVID-19 pandemic.

## Background

Many medical students in high-income countries in the Global North participate in medical electives related to global health in low- and middle-income countries, also known as the Global South. These electives normally have a short duration, such as four weeks, during which the visiting students study alongside students in their host institutions and live in communities close to the host institutions to familiarize themselves with the culture of the host country. The objectives of the students participating in these electives include improving their medical and surgical skills, improving their language skills, and gaining in-depth knowledge of infectious diseases [1].

Makerere University College of Health Sciences, an institution of higher education, has established partnerships with several other institutions worldwide, including the University of Brescia and “*Magna Græcia*” University, which have established a partnership with the main objective of student exchange. As part of this partnership, one hundred and fourteen students and 2 residents received clinical placements in the main teaching hospital of Makerere School of Health Sciences and its affiliated sites of Kawempe and Kiruddu in Kampala, Uganda. In addition, several students received placements with community-based education research services such as Buluba Hospital and others. Through these efforts, students from Italy have completed clinical rotations to fulfil their global health electives.

The objective of this article is to comment on the preparation of these students for away rotations. In addition, the article discusses the peculiarities of student exchange programmes during the SARS-CoV-2 pandemic.

## Attitudes and difficulties of students undergoing training in resource-limited settings

Students returning from the Global South after completing their electives often declare that the experience has changed their perspective on their profession and, occasionally, their life: students noted that the elective led to numerous attitudinal changes, such as a greater appreciation of the importance of public health, health service delivery, cross-cultural communication, and the challenges of providing health care to underserved communities. Additionally, these electives are associated with a reported increase in the availability of knowledge and training on tropical diseases, suggesting that these physicians with field experience in global health may be more competent in treating imported diseases in immigrants and travellers who have returned from the Global South [2]. These experiences allow such physicians to consider their personal and professional identities. One study identified transformative learning as a possible mechanism linking the individual to the process of professional identity formation via the elements of a disorienting experience, an emotional response, critical reflection, a change in perspective, and a commitment to future actions [3].

Working in countries with limited resources, however, requires specific skills, both emotional and technical, that must be acquired before departure. The ethical relevance of training students in countries with limited resources is related to the need to avoid risks both for both the physicians and the patients [4]. In fact, the well-known risk of “humanitarian neo-colonialism” in the field of welfare and research [5] must be mitigated with the application of the principles of the *cura te ipsum* (heal thyself) and *primum non nocere* (first, do no harm). Modern medicine has been viewed as an artefact of colonialism because the science underlying modern medicine emerged from Western knowledge structures based on a history of colonialism. Researchers have suggested that the colonial roots of modern medicine rooted in the West must be reexamined [6].

While the task of ensuring a traineeship experience in countries with limited resources, as opposed to merely transmitting the theoretical knowledge of problems and their possible solutions, seems to be important, those who travel to countries with limited resources to participate in a traineeship activity must also be adequately trained with regard to the following points: knowledge of the culture, customs, habits and language(s) of the host country and the development of a nonjudgmental approach to health issues and practices in the host country.

One student in the final year of the course of studies in medicine and surgery (R.S.) wrote the following after returning from the joint training programme conducted by the “*Magna Græcia*” University of Catanzaro (Italy) and the University of Makerere, Kampala (Uganda):

*“AIDS, hepatitis, tuberculosis; almost everyone in the hospital suffers from them, and even if you have not been infected, the fear of being able to get infected by these infections in some way creeps inside you, and then you are mechanical, attentive to everything: mask and a double pair of gloves. And you are constantly watching and listening.*

*The strong smells get into your head (…). Drugs are scarce, as are gauze and gloves. The medical staff is very limited, and the patients must wait for a long time; the wait is long, but there are no protests, and even the relatives squatting on the ground are impassive. A dying child receives no treatment. There’s nothing more we can do for him. A young doctor in his thirties notices something in your eyes; he approaches you and whispers ‘this is Uganda’.*

*And then you finally understand the words of your professor, who insisted that you should not judge anything you would see. You understand, but it’s hard not to do so”.*

Knowledge of and experience with industrialized countries are difficult to transfer to countries with limited resources. An emblematic example of this difficulty pertains to the skills and techniques that are necessary in cases of emergency, especially in territories outside equipped centres (i.e., so-called “wilderness medicine”). In this context, it is necessary to apply the principles of multifunctionality (the appropriate use of the same tool for different procedures) and cross-functionality (the use of nonspecific tools for a given medical act when specific tools are unavailable), which are illegal in industrialized countries. The true value of training in wilderness medicine lies in the development of highly refined, system-oriented thinking on the part of regular emergency care providers working in limited resource environments. For this reason, such training should at least be considered for inclusion in training programmes [7].

It is also necessary to react in a psychologically appropriate way to the problems encountered while performing activities in “difficult” contexts when faced with overwork or conditions of risk; these problems may even pertain only to climatic differences, a lack of resources or the incongruity of the health system with standard practices in one’s country of origin, or the impact of the frequently severe suffering and death of patients, which can result in a high risk of burn-out among physicians (self-reliance and resilience).

*“The work is hard. The food is not great, and you’re still afraid to eat most things. Sometimes, after a few days, “Montezuma’s revenge” falls on you inexorably, you know. Don’t worry too much, it’ll just be only for a couple of days.*

*Time goes by, and you wonder how it’s been 20 days without you realizing it; there’s so many things you’ve done. You’re starting to settle in, you’re more casual. Small or large animals don’t bother you anymore.*

*In the end, the food is not so bad; in fact, the beans are excellent as is the chicken; the fruit is the best you’ll ever eat. You start putting aside your fears. You really know the locals, what great people! Not all of them, of course. You spend a lot of time looking at the details, the little things you used to miss. If you smile, everyone smiles. Make friends. You went to help, but so far, it’s everybody else helping you.*

*The hospital’s getting better and better, paranoia’s a memory or something, there’s so much to do. Be fascinated by the skill and preparation of the local doctors and students. With what little they have, they can do so much. Being accustomed to the shortage of drugs and materials has made improvisation an art. Their genius leaves you amazed”.*

Another relevant aspect is the linguistic preparation of students in away rotation programmes. Adequate preparation facilitates better and faster learning, thus enabling students to integrate into the country they visit in the shortest possible time by immediately immersing them in the social and professional cultural context of the place in question.

Medical schools should start to seriously consider the development of a more structured medical elective, and if this task is impossible due to time and resource constraints, many other steps can be taken to improve the quality of predeparture training and awareness.

To ensure that international medical elective programmes can be effective and enriching for all parties concerned, medical schools and programme coordinators should ensure that students’ educational, health and safety, ethical and social responsibility needs are met through strategic site visits, comprehensive predeparture training programmes, travel health assessments, and debriefing and health screening sessions after their return [8, 9].

Preparing students for this type of experience is of fundamental importance. Accordingly, a course providing training in global health is necessary and should be compulsory for those wishing to participate in an elective programme. Adequate training is also necessary to make students aware of the difficulties they will face, to assess their motivation and to prepare them for the setting in which they will work. Similarly, knowledge of the language of the country to which they will travel is essential.

Furthermore, sharing and comparing experiences with students or health professionals who have had this experience previously is essential, and meetings and events involving international coordinators can be very useful. Moreover, we noted that comparing experiences with students from universities in other countries involved in mutual exchange programmes can be very beneficial. For example, this year, at the University of Catanzaro, students from Makerere University in Kampala, Uganda, delivered lectures to students who would be participating in an elective programme at the same university.

Indeed, elective programmes in which medical students from low- and middle-income countries travel to high-income countries should be developed to ensure mutually effective results. These programmes are very important to the task of creating and strengthening more structured collaborations that can benefit both sides. They can also be considered an integral part of the training of students who participate in medical electives, for example, by encouraging visiting students to given lectures on the most common diseases in their countries and the corresponding differences in diagnosis and treatment. This year, two students from Makerere University, Kampala, Uganda participated in elective study at the “*Magna Græcia*” University of Catanzaro, Italy. As one of these students (K.N.) wrote upon his return home,

*“It was one of the most insightful and crucial experiences in my academic journey thus far, based on the fact that I learnt a lot about practice in a resource-rich setting to prepare me for future real-world work. I had several objectives, most of which were achieved. They included understanding the diseases and their management in a resource-rich setting, observing technological advances and other aspects of the Western health system first-hand and learning how they can be incorporated into the Ugandan health care system. I was interested in making a global community of friends, learning the Italian language and culture. […] I participated in academic ward rounds, presentations at seminars, and a clinical clerkship”.*

## How can students be prepared for global health?

Institutions of higher education in high-income countries must prepare their medical students for global health electives in the Global South. Students must be prepared to develop a mindset of learning rather than merely helping, and they must understand the culture of the host institution in the Global South to comprehend the manner in which patient care is provided in a resource-limited setting.

This preparation of students should be facilitated by training on health problems caused either directly or indirectly by transnational factors [10]. According to a transnational perspective on social justice, global health highlights inequalities in the quality of health care both within and across countries. Given the complexity of this field, global health requires a transdisciplinary and multimethodological approach that makes use of the contributions of both the social and human sciences as well as the natural and biomedical sciences.

Moreover, the objectives of global health care are practical and aim at generating real changes in health professionals, communities and societies to address the gap between scientific evidence and operational decisions, a process which is defined in the literature as the “*know-do gap*” (http://www.who.int/kms/events/Know_do_gap_APablos.pdf). Additionally, the adoption of problem-based learning curricula can help reduce the “*know-how gap*” by encouraging students to play a more active role in their own training, helping them improve their critical thinking skills and promoting collaboration both among themselves and with the teaching body [11]. The goal of preventing more than eight million victimizations due to poor health care can also be achieved by making a joint effort to improve education in global health [12].

*“Many die every day, but you can’t get used to it. You shake hands with a father; he just lost his son in a car accident, but in the end, he consoles you: “He’s gone to a better place than this,” he says. In the evening, you go home (that accommodation that you used to walk around in with suspicion has already almost become your home), and after all, you are happy. You didn’t do anything, you put in a few stitches, you oversaw a couple of withdrawals and administered a few injections, you helped someone eat, you played with a baby so he wouldn’t cry, you changed bandages, you assisted a girl in her final moments of life. You are satisfied with yourself, maybe for the first time, really (…) You come home, and you think that this basically means being a doctor”.*

Knowledge of global health and optional traineeship programmes should be included in curricula for medical students and health professionals (http://globalhealth.thelancet.com/2016/07/20/rethinking-undergraduate-global-health-education-bellagio-global-health-education). Indeed, global health education should respond to current priorities in global health. A key point to consider is the fact that the movement to promote global health will inevitably require greater input from disciplines outside the fields of medicine and public health. While there is still demand for training in specialized areas such as tropical medicine, in the future, three types of global health doctors should be educated: the globalized doctor (who is primarily focused on their own health system), the humanitarian doctor and the policy doctor will all become recognized and valid categories. Therefore, training must be predicated on the broader social, economic and political aspects of global health. This approach must be complemented by a critical reflection on the perspectives and values that underlie ‘global health’ as a necessary part of students’ own professional development, whether they continue to work at home or abroad [13]. The COVID-19 pandemic further highlighted the importance of the promotion of universal health coverage with better governance and connections to social protection systems [14].

Many studies have shown that medical students are very interested in the various epistemological issues that underlie global health and the ways in which global health is fundamental for understanding contemporary health care and health even in the Western world. In fact, many students view this field as an essential aspect of working in the globalized world, which is therefore fundamentally important to all health care professionals [15].

Given major global changes and the corresponding increasing interest in global health, the need for integrated and formalized programming that allows students to develop relevant skills as well as the ability to apply theoretical concepts in high-impact global health initiatives is growing. Several examples demonstrate that a structured global health capstone can be used to teach global health skills and prepare students for careers as global health professionals and leaders [16].

Taking global health courses in medical school has been associated with a significant increase in the likelihood of pursuing a career in which the individual works with underserved populations. Individuals who have participated in these courses have reported multiple benefits, particularly improved cultural humility and in-depth knowledge of public health [17].

Thus, this type of training is critically important not only for individuals who decide to gain experience in resource-limited countries but also in general as a fundamental component of medical education in the modern world.

Medical students recognize the benefits of including global health topics in the medical curriculum as well as incorporating international clinical rotations into their training. Many studies have shown that students who have completed a rotation in a developing country report increased skills and confidence, greater sensitivity to cost issues, less dependence on technology, and a greater appreciation for cross-cultural communication. They learn to practice medicine with limited access to lab tests and expensive diagnostic procedures, they rely on greater skill in physical examination and depend less on lab values, radiology, and other diagnostic tests, they develop a deeper appreciation for issues related to global public health and they become more culturally sensitive [18].

A critical review of the concept of global health and the associated teaching is also necessary. Indeed, global health often involves partnerships between institutions in low- and middle-income countries that were previously colonized and high-income countries that were colonizers. Little attention has been given to the legacy of these former colonial relationships and the influence they have had on global health initiatives. There have been recent calls to decolonize global health education and reexamine the assumptions and practices underlying global health partnerships. In addition, research partnerships tend to benefit the partner with the best resources [19].

Health care in the contemporary environment involves a set of complex and interrelated issues: pandemics, international conflicts, climate change, economic crises, rising health care costs, increasing poverty and disparities, and large-scale migration. The skills that can be developed by reference to the knowledge and practices of the field of ‘’global health’’ are becoming necessary not only for health professionals in resource-limited countries but also for those in high-income countries. These skills allow the individual to understand local, national, and international scenarios and the interconnections among them to make use of cultural and intercultural competencies that address global health needs, thereby promoting population health effectively and equitably [20, 21].

Human migration is now at an all-time high of 240 million people, a situation which shapes world events and leads to public debates. If migrant health is addressed properly, this situation can lead to profound benefits for the individual, for the entire populations of countries of first asylum and resettlement countries, and for global health security. Barriers to accessing health care arise at the levels of the patient, the doctor, and the health care system. At the individual or patient level, disease-related stigma, poverty, discrimination, and linguistic and cultural challenges can lead to underdiagnosis of disease and the uptake of treatment. Barriers at the level of the doctor, such as limited knowledge of migrants’ health needs and difficulties communicating, can be very problematic. The increasingly global and mobile world requires all health professionals to be aware of the health needs of the migrant population, as they are likely to encounter migrants in their practice. Health workers should be aware of the geographical distribution of diseases and risk factors for specific infectious diseases. They must be aware of “rare and tropical infections” related to migration and return travel and of the fact that migrants may harbour multiresistant organisms, which has important implications for hospital infection control practices [22, 23].

Another key point pertaining to the necessity of integrating global health into the curricula of health care professionals is the need to address the emerging needs of travel medicine, which is still based on a strongly Western perspective. In 2015, for the first time in history, emerging economies such as China, India and Brazil reached parity with the traditional continents of North America and Europe in terms of travel volume. Projections show that by 2030, these countries will overtake so-called Western countries, including North America, Europe, Australia, New Zealand, and Japan. Asia, the Pacific, the Middle East, Africa, and Latin America are now increasingly the source of travellers, rather than merely the countries that receive travellers. These changes will also result in different patterns of geographical dispersion of infectious diseases [24].

Finally, the economic crisis that led to an increase in health inequalities and the corresponding increase in health care costs due to numerous factors, primarily the increase in the world population and rising average ages, cause health specialists to be faced with a shortage of resources even in industrialized countries. Adequate training in the Global South facilitates the acquisition of the skills and understanding necessary to manage diagnostic work-up and therapy with minimal resources to promote the best interests of the individual patient and the community, a lesson that is currently useful even in the Global North with respect to improving the cost-effectiveness of health interventions.

Overall, the inclusion of away rotations as a key experience in global health are crucial to the training of physicians. With this objective in mind, certain characteristics defined by the CanMEDS framework should be considered carefully. According to the CanMEDS framework, a medical expert should contribute her or his expertise and influence while working with communities of patient populations to improve health, working with those whom they serve to determine and understand their needs, speak on behalf of others when necessary, and support the mobilization of resources to effect change. In other words, medical experts have the ability to contribute their expertise to public health as health advocates. Simultaneously, they should be professionals who reflect the expectations of contemporary society regarding physicians, including by exhibiting clinical competence, a commitment to professional development, the promotion of the public good, and adherence to ethical standards and values such as integrity, honesty, altruism, humility, and respect. It is also recognized that to provide optimal patient care, physicians must take responsibility for their own health and that of their colleagues. These skills contribute to the development of a medical expert.

Unfortunately, in general, Italian universities rarely acknowledged the importance of global health in curricula for medical students. However, an attempt to promote the teaching of global health was initiated in 2010 by the Italian Network for Teaching Global Health, which offered interesting activities and online courses (http://www.educationglobalhealth.eu/it/riisg). Moreover, a structured collaboration between the Ministry of Foreign Affairs and International Cooperation and the Conference of Rectors of Italian Universities has been ongoing for years; the aim of this collaboration is to implement and promote a culture of cooperation in Italian universities through the development of specific programmes (http://www.istruzione.it/archivio/web/universita/cooperazione-internationale.html).

Appropriate training is a basic requirement for individuals who aspire to play a health-related role in the international arena. On its website, the Italian Agency for Development Cooperation publishes a list of the courses and master’s degrees available (https://www.aics.gov.it/home-ita/opportunita/altre-opportunita/formazione). Although global health education has received increasing attention, a situation which is in alignment with the vision of the Italian Network for Global Health Education, which offers several courses on global health throughout Italy, more academic commitment is needed to mandate the inclusion of global health in the curricula of medical schools and other health faculties [25].

On what basis can a decision be made among the available courses? In an attempt to answer this question, several orientation sites for young people have recommended focusing on the following aspects: (1) seniority of the training proposal, e.g., the number of years for which the course or programme has existed; (2) the presence of mixed teaching staff because it is important that professionals working in the field are present as members of the faculty in addition to professors from the academic world; (3) success indicators, such as the placement rates of students, i.e., the number of students who are able to find a job in their specific field of interest and the length of time receiving a degree is required to be placed; and (4) the guarantee of practical experience in the field, which should be integrated into the activities of the course, such as internships in resource limited settings (even in the context of short courses).

Several traineeship programmes that use innovative learning platforms to focus on specific problems are available. These programmes are based on partnerships between institutions in resource-constrained countries and those in industrialized countries, for example, “Afya Bora“[26]. Consortium aims to teach information regarding the prevention and treatment of HIV/AIDS infections and offers interprofessional training using several complementary teaching methodologies, including online learning. The course materials are available online (http://afyaboraconsortium.org/new/materials.html#modules). The University of Brescia also offers an international postgraduate course in global health within the Troped network, which was developed more than 25 years ago (www.troped.org).

## The impact of the COVID-19 pandemic on the exchange programmes

The final aspect that must be considered is that conventional medical education and international exchange programmes have been severely threatened by the COVID-19 pandemic. Although obvious difficulties emerged, the task of coping with these problems should be viewed as a real opportunity to contribute to the advancement of medical education through active curricular innovation and transformation as well as a key moment for many disciplines of medicine [27]. International medical elective programmes have occasionally been discontinued; however, it is very important for these programmes to be restarted safely and effectively since vaccines are available and since our knowledge of how to prevent and treat SARS-CoV-2 infection has improved dramatically. Additionally, it is important to emphasize the fact that student-led engagement in the pandemic response represents a unique opportunity for medical educators and students to engage in practice-based learning with respect to preparedness and pandemic response. It is imperative for academic medical institutions to seize this opportunity to identify effective ways of preparing future physicians to support robust public health responses [28].

A student from Makerere University (C.P.N.) wrote the following after spending time at the teaching hospital of “*Magna Graecia*” University of Catanzaro:

*“In the infectious diseases rotation, because of the scourge of the pandemic, the unit at Mater Domini Hospital had to be transformed into an entirely COVID treatment unit. Due to the fact that, back home in Makerere, we were not allowed to interact with COVID patients for safety reasons, this would be our first-time interfacing with them, and it was nerve wracking! Slowly I came to learn that there was nothing to be afraid of because the personal protective equipment was always available in abundance and very stringent measures had been put in place to ensure that there would be no contamination and therefore reduced chances of infection. These included proper demarcation of the clean “pulito” and dirty “sporco” parts of the unit where one needs to be fully dressed in protective gear, and full disinfection was necessary before crossing from end to end. We were also subjected to mandatory COVID tests just as an extra measure to avoid infection of the other medical staff.*.

*Aside from this, my experience in both disciplines was splendid. I had the opportunity to fully appreciate what goes into caring for a COVID-19 patient from the time of admission to the time of discharge. I had not expected to have much physical contact with the patients, but to my great surprise and enjoyment, we were allowed to perform certain procedures”.*

These experiences caused us to realize the important of involving the students in this challenge, constantly taking safety precautions, and allowing them to operate on the frontlines. Due to this involvement of students in real clinical practice, elective programmes play a fundamental role in the pandemic era by preparing students for the difficulties that they must face in their future as medical professionals wherever that future will take place [29].

*“The situation created by the pandemic is likely permanent, and as the medical fraternity, we must continue to adapt and create ways for students to learn and participate in patient care; this is the only way in which we will be able to make any kind of real progress”.*

## Conclusion

Preparing health workers to work in developing countries is a teaching experience that develops specific skills and also takes place “in the field”; it is multidisciplinary and transcultural. The foundation for this preparation should be established by courses on medicine and surgery and in other health-related professions. In our experience, students who have had the opportunity to become motivated to continue following the path of international collaboration appreciate methods of teaching that feature “field” experiences. In an increasingly globalized world in which human migration is at an all-time high, a world that includes the COVID-19 pandemic, increasing inequalities and especially the increase in travellers from resource-limited countries, structured medical electives and preparation for global health are a key part of the training of future health professionals. The educational models and opportunities available in this field are constantly evolving [30] and deserve to be monitored and disseminated in both the academic and institutional fields as well as through voluntary associations. The following is a final thought expressed by one Italian student after his experience at Makerere University:

*“It’s the last day; say hello to everyone and take the usual pictures. You’re happy to see your loved ones again, but then you think you were enjoying that place, you made friends, the hospital started to trust you, you were becoming one of them and, oddly enough, you don’t want to leave anymore. And yet, you think you’re going to come back, no matter what. Now you can’t help it”.*

## Data Availability

Not applicable.
